# Enhanced ZnR/GPR39 Activity in Breast Cancer, an Alternative Trigger of Signaling Leading to Cell Growth

**DOI:** 10.1038/s41598-018-26459-5

**Published:** 2018-05-25

**Authors:** Hila Ventura-Bixenshpaner, Hila Asraf, Moumita Chakraborty, Moshe Elkabets, Israel Sekler, Kathryn M. Taylor, Michal Hershfinkel

**Affiliations:** 1 Department of Physiology and Cell Biology and The Zlotowski Center for Neuroscience, Faculty of Health Sciences, Ben-Gurion University of the Negev, Beer-Sheva, Israel; 2 The Shraga Segal Department of Microbiology, Immunology and Genetics, Faculty of Health Sciences, Ben-Gurion University of the Negev, Beer-Sheva, Israel; 3 Breast Cancer Molecular Pharmacology Group, School of Pharmacy and Pharmaceutical Sciences, Redwood Building, Cardiff University, King Edward VII Avenue, Cardiff, CF10 3NB UK

## Abstract

Acquired resistance to the estrogen receptor (ER) antagonist tamoxifen, is a major obstacle in treatment of breast cancer. Changes in Zn^2+^ accumulation and distribution are associated with tamoxifen-resistance and breast cancer progression. The Zn^2+^-sensing G-protein coupled receptor, ZnR/GPR39, triggers signaling leading to cell growth, but a role for this receptor in breast cancer in unknown. Using fluorescence imaging, we found Zn^2+^-dependent Ca^2+^ release, mediated by ZnR/GPR39 activity, in TAMR tamoxifen-resistant cells derived from MCF-7 cells, but not in ER-expressing MCF-7 or T47D cells. Furthermore, ZnR/GPR39 signaling was monitored in ER negative BT20, MDA-MB-453 and JIMT-1 cells. Expression of ZnR/GPR39 was increased in grade 3 human breast cancer biopsies compared to grade 2. Consistently, analysis of two breast cancer patient cohorts, GDS4057 and TCGA, indicated that in ER-negative tumors higher ZnR/GPR39 mRNA levels are associated with more aggressive tumors. Activation of ZnR/GPR39 in TAMR cells triggered MAPK, mTOR and PI3K signaling. Importantly, enhanced cell growth and invasiveness was observed in the ER negative breast cancer cells, TAMR, MDA-MB-453 and BT20 cells but not in the ER expressing MCF-7 cells. Thus, we suggest ZnR/GPR39 as a potential therapeutic target for combination treatment in breast cancer, particularly relevant in ER negative tumors.

## Introduction

Activation of signaling pathways and transcription by the steroid hormone estrogen, via the estrogen receptor (ER), regulates mammary epithelial cell growth. In breast cancer, the expression of ER is used as a biomarker to guide therapy, and ER positive breast cancer patients are often treated with antihormones such as tamoxifen. However, resistance of tumors to tamoxifen develops in the majority of treated patients, leading to recurrence and progression of the disease^1,2^. Tamoxifen resistance may occur through alteration of different signaling pathways, for example, upregulation of EGF, IGF and HER2 receptor tyrosine kinases may downregulate ER expression^3,4^. In addition, acquired mutations in the ER have been shown to induce endocrine resistance^5,6^, and early identification of these mutations can guide therapy switching^6,7^. Constitutive activation of intracellular signaling, associated with cell growth, plays an important role in cancer progression and aggressiveness, particularly prominent is the PI3K/AKT pathway that is activated in 75% of breast cancers^8^. Indeed, inhibitors of the PI3K/AKT pathway are proposed as single agent drugs, or, more effectively, in combination treatment with ER inhibitors^9–13^. Revealing mechanisms that underlie acquisition of tamoxifen resistance or constitutive signaling, is essential to elucidating novel therapeutic approaches to breast cancer.

Zinc is an essential micronutrient, and free Zn^2+^ ions emerged as important cellular signaling molecules involved in cell growth and survival^14,15^. Changes in Zn^2+^ levels and Zn^2+^ homeostatic proteins are monitored in breast cancer cells and tissues and are associated with more invasive behavior^16–20^. Activation of kinase signaling pathways in breast cancer MCF-7 cells is mediated, for example, by the endoplasmic reticulum Zn^2+^ transporter ZIP7^21,22^. Increased expression of ZIP7, concomitant with endoplasmic reticulum Zn^2+^ accumulation, was monitored in tamoxifen resistant cells derived from MCF-7 cells, termed TAMR^22–24^. These changes in ZIP7 expression were further associated with enhanced EGFR activation and breast cancer cell growth^25^. Furthermore, changes in the expression of different members of the ZIP family of Zn^2+^ transporters lead to epithelial to mesenchymal transition in breast cancer cells^20,26–28^. In normal breast tissue, Zn^2+^ is transported by ZnT2 into the milk-containing vesicles^29^. In breast cancer cells and tissues, downregulation of ZnT2 induces mislocalization of cellular Zn^2+^ leading to cell survival^16^, likely via attenuation of lysosomal cell death mechanisms^30,31^.

Free-Zn^2+^ concentrations, within the cytoplasmic region or extracellular domain, are in the femtomolar range, but this ion is found in high concentrations in vesicular organelles in many cell types^32^. The release of vesicular Zn^2+^ induces robust and transient rises in its local concentrations, followed by rapid re-uptake via ZIP transporters or chelation by Zn^2+^ binding proteins^15^. Such transient changes in concentrations of extracellular Zn^2+^ induce signaling via a Zn^2+^-sensing, G-protein coupled receptor, ZnR/GPR39^33–35^. The ZnR/GPR39 triggers intracellular Ca^2+^ release and subsequently activates the mitogen activated protein kinase (MAPK) or PI3K/AKT pathways^36–38^. Indeed, Zn^2+^-dependent activation of MAPK pathway in keratinocytes was mediated by ZnR/GPR39 and induced enhanced cell growth in a scratch assay model^39^. Similarly, ZnR/GPR39 activation of MAPK, PI3K and clusterin were shown to enhance survival of colon cancer cells following treatment with apoptosis-inducing butyrate^40,41^. The ZnR/GPR39-dependent epithelial cell growth is mediated by the signaling pathways that are constitutive active in tamoxifen resistant breast cancer^8,42^. We, therefore, hypothesized that ZnR/GPR39 may be an upstream regulator of breast cancer cell proliferation.

## Results

### ZnR/GPR39 is functional in breast cancer cells

**Figure 1:**
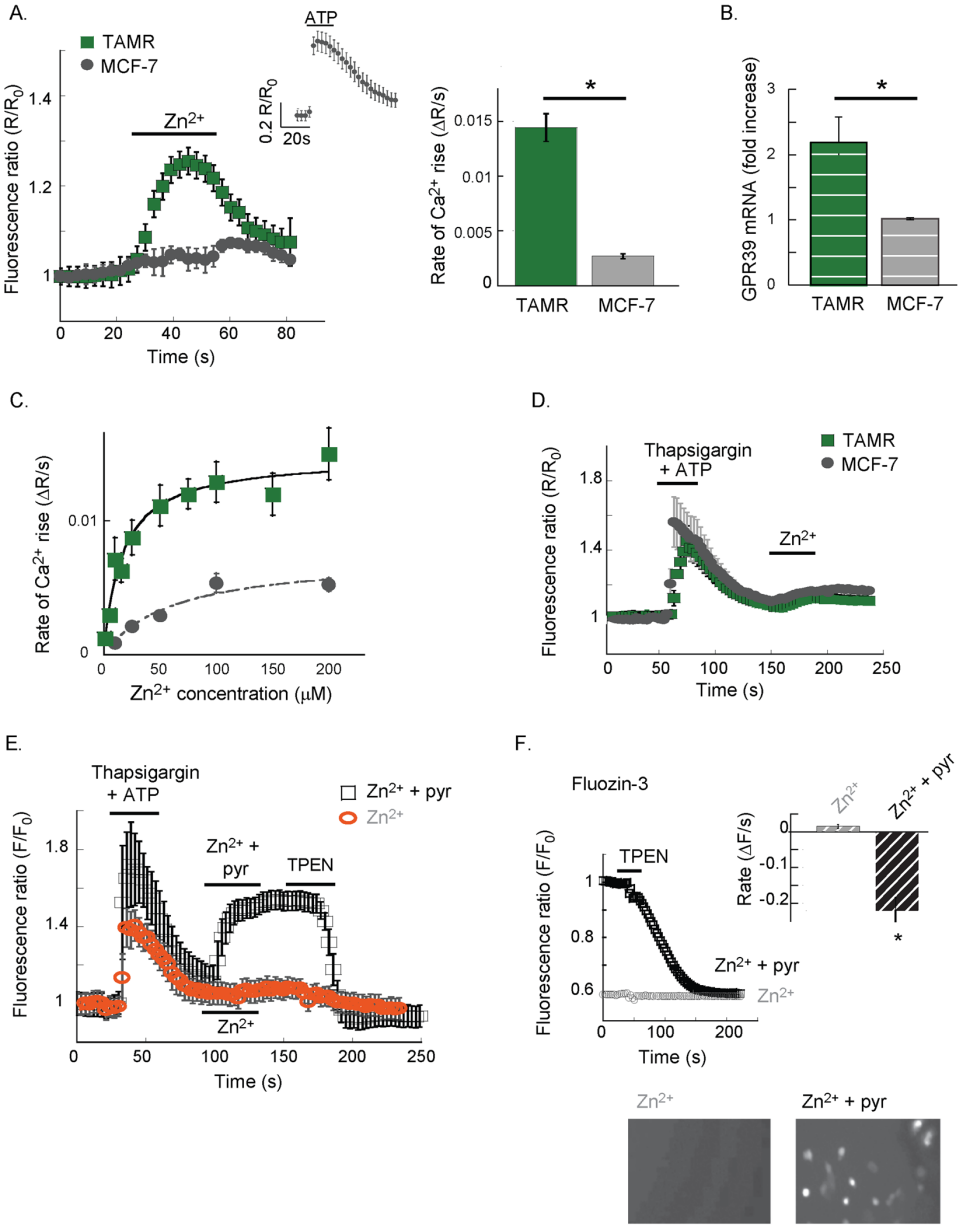
Extracellular Zn^2+^ triggers Ca^2+^ responses in TAMR but not in MCF-7 cells. (**A)** TAMR or MCF-7 cells, loaded with Fura-2, were imaged, and Zn^2+^ (200 μM) was applied at the indicated time. Representative Ca^2+^ response averaged over 10–15 cells is shown (left panel). The average rate of signal rise is shown in the bar graph (right panel, n = 5 slides for each condition in 4 independent experiments, p < 0.05 t-test). Inset shows a representative Ca^2+^ response triggered by the purinergic metabotropic receptor agonist, ATP (25 μM). (**B**) Quantitative PCR analysis of GPR39 mRNA levels in the MCF-7 versus TAMR cells (n = 3, p < 0.05 t-test). (**C**) Dose response analysis of the Zn^2+^-dependent Ca^2+^ response in Fura-2 loaded cells was performed, using the paradigm described in A, for TAMR and MCF-7 cells (n = 4, p < 0.05 t-test). (**D**) Using thapsigargin (200 nM) and ATP (25 µM), as indicated, the intracellular Ca^2+^ stores were depleted, and subsequent baseline fluorescent signal indicated removal of cytoplasmic Ca^2+^. Then, Zn^2+^ was applied as in A; representative traces from TAMR and MCF-7 cells are shown. (**E**) Ca^2+^ stores were depleted as in D., and Zn^2+^ (200 μM) was applied in the presence (black) or absence (orange) of the ionophore pyrithione (5 μM). The cell permeable chelator TPEN (10 μM) was finally added. Representative response of Fura-2 signal is shown. (**F**) Cells loaded with the Zn^2+^-sensitive dye, FluoZin3, were pre-treated with Zn^2+^ alone or in the presence of pyrithione (5 μM, 2 min). Cells were then imaged and TPEN (10 μM) was applied, representative traces are shown. Averaged rates of fluorescence decrease are shown in the insert (n = 5 coverslips in 4 independent experiments, p < 0.05 t-test). Bottom panel shows representative images of FluoZin-3 fluorescence in cells pre-treated with Zn^2+^ alone or in the presence of pyrithione (5 μM, 2 min).

We first asked if there is Zn^2+^-dependent Ca^2+^ signaling in breast cancer cell lines, initially comparing the response of MCF-7 cells (ERα, PR positive cells that express low levels of HER2) to that of the tamoxifen resistant TAMR cell line derived from MCF-7 cells^25,43,44^. Extracellular Zn^2+^ (200 μM) triggers Fura-2 responses in TAMR cells but not in MCF-7 cells, which have lower levels of ZnR/GPR39 mRNA (Fig. 1A,B). Application of ATP (25 µM), which activates the purinergic metabotropic pathway, triggered a clear response in MCF-7 cells, indicating that the IP3 pathway and Ca^2+^ intracellular stores are intact in these cells (inset Fig. 1A). Dose response analysis (Fig. 1C) of the Zn^2+^-dependent Ca^2+^ response, indicates that TAMR cells have a Km of 19 ± 8 µM to Zn^2+^, while MCF-7 show only residual activity with Km of 43 ± 19 µM and maximal Ca^2+^ signaling activity that is 3-fold lower than that of TAMR cells. To determine the role of the IP3 pathway in the Zn^2+^-dependent Ca^2+^ response, intracellular Ca^2+^ stores were depleted, using the SERCA inhibitor Thapsigargin (200 nM) and ATP (25 µM). Subsequently, Zn^2+^ did not trigger a response in either TAMR or MCF-7 cells (Fig. 1D), suggesting that the Zn^2+^-dependent response requires intact intracellular Ca^2+^ stores. Since Fura-2 is also sensitive to Zn^2+^, we asked if the fluorescence response monitored in TAMR cells may be also triggered by Zn^2+^ permeation. Following depletion of the Ca^2+^ stores, as above, cells were treated with Zn^2+^ (200 μM) in the presence or absence of the Zn^2+^ ionophore, pyrithione (5 μM, Fig. 1E). The Fura-2 fluorescence rise was only monitored in cells treated with Zn^2+^ in the presence of pyrithione (Fig. 1E) and subsequent addition of the cell permeable Zn^2+^ chelator, TPEN (10 μM), lowered this signal. Similarly, cells loaded with the Zn^2+^-sensitive fluorescent dye Fluozin-3 showed increased fluorescent signal only when Zn^2+^ was applied in the presence of pyrithione (Fig. 1F), and TPEN reversed this signal. Thus, we conclude that extracellular Zn^2+^ triggers release of intracellular Ca^2+^ from thapsigargin sensitive stores in TAMR cells.

**Figure 2:**
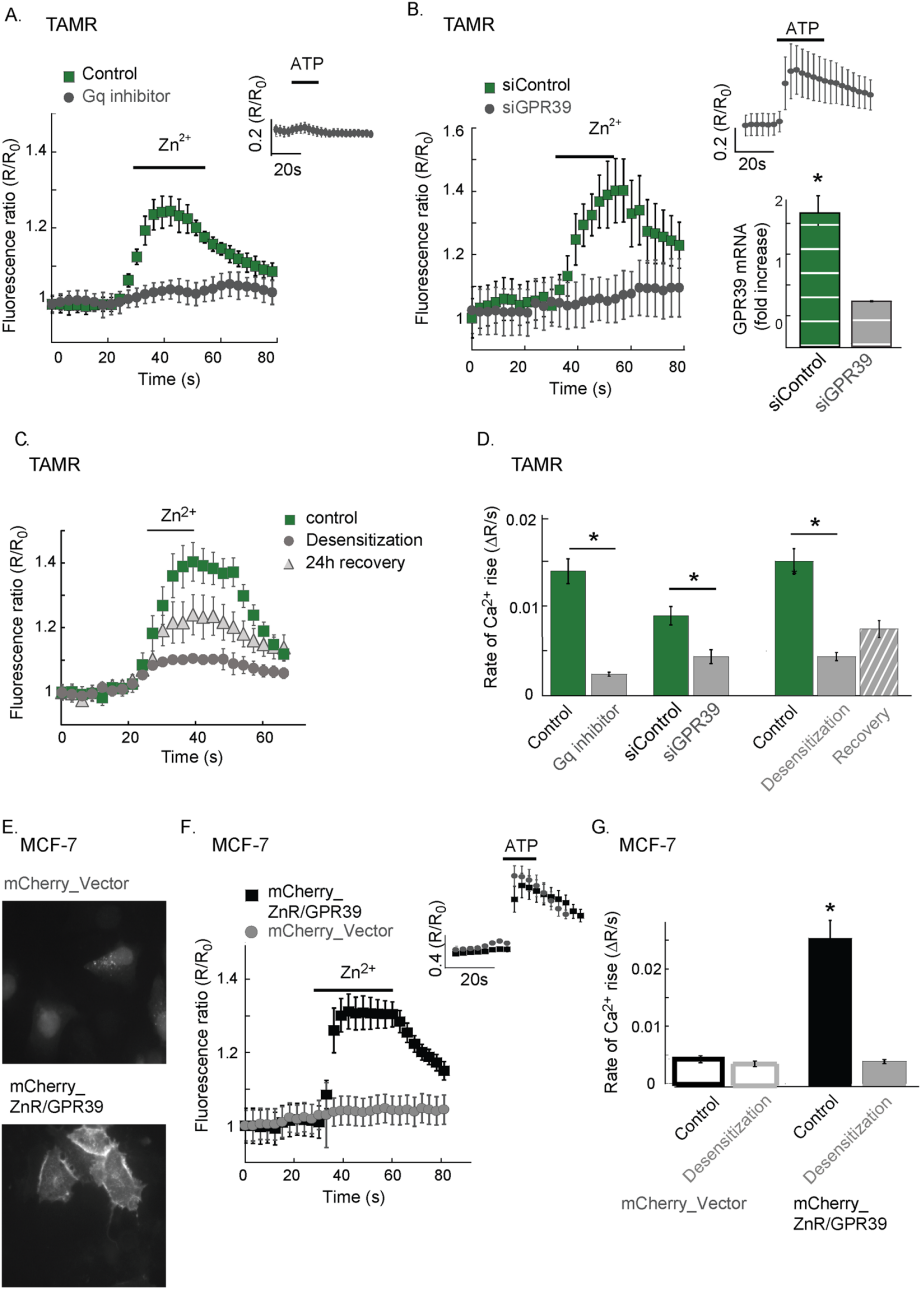
ZnR/GPR39 mediates Zn^2+^-dependent intracellular Ca^2+^ signaling in TAMR cells. (**A**) Representative Fura-2 fluorescent signal of TAMR cells pre-treated with the Gαq inhibitor, YM-254890 (1 μM, 5 min) or without it (control). At the indicated time, Zn^2+^ (200 μM) was applied. Inset shows the Ca^2+^ response of YM-254890 treated cells to the purinergic agonist ATP (25 μM). (**B**) Representative Ca^2+^ response of TAMR cells transfected with siGPR39 or a scrambled control (siControl) to Zn^2+^ (200 μM, as indicated, left panel). Right panel (top) shows the response of siGPR39 cells to the purinergic agonist ATP (25 µM). Right panel (bottom) depicts mRNA level of ZnR/GPR39 in siGPR39 and siControl cells (n = 4 coverslips for each condition, in 6 independent experiments, p < 0.05 t-test). (**C**) TAMR cells were pre-treated with lower concentration of Zn^2+^ for a prolonged time (100 μM for 25 min, 37 °C). Cells were then maintained in Zn^2+^-free Ringer’s solution for 30 min (desensitized) or returned to the incubator for 24 h (recovery, n = 4 coverslips), and subsequently ZnR/GPR39 activation was monitored using the paradigm described in Fig. 1. Representative Ca^2+^ signals in control cells, desensitized cells and cells following recovery are shown. (**D**) Average rates of initial Ca^2+^ response as determined from A-C. (n = 4 coverslips for each condition, in 6 independent experiments, p < 0.05 t-test). (**E**) Fluorescent imaging of MCF-7 cells transfected with an mCherry- vector or mCherry-ZnR/GPR39. (**F**) Representative Fura-2 signals, indicating Ca^2+^ responses, of MCF-7 cells transfected with the mCherry-vector or mCherry-ZnR/GPR39, following application of Zn^2+^ (200 μM, at the indicated time). Inset shows the purinergic response of the mCherry-vector cells, indicating that the metabotropic pathway is intact in these cells. (**G**) Cells expressing mCherry-vector or mCherry-ZnR/GPR39 were desensitized using the paradigm described in C. After 30 min in Zn^2+^-free Ringer’s solution, Fura-2 loaded cells were imaged and the initial rate of the response to Zn^2+^ (200 μM) was determined. The average of the initial rate of the Zn^2+^-dependent Ca^2+^ response in desensitized cells or controls is shown. (n = 5 coverslips for each condition, in 4 independent experiments, p < 0.05 ANOVA).

We then asked if a Gαq-dependent signaling pathway is mediating the Zn^2+^-dependent Ca^2+^ release in TAMR cells^37,38^. In the presence of the Gαq inhibitor, YM-254890 (1 μM), the Zn^2+^-dependent Ca^2+^ response was inhibited (Fig. 2A,D) as well as the purinergic receptor signaling triggered by ATP (see inset). We then specifically addressed the role of ZnR/GPR39 in mediating the Zn^2+^-dependent Ca^2+^ response in breast cancer cells by either overexpression or siRNA silencing of GPR39 (siGPR39). TAMR cell transfected with the siGPR39 construct, exhibited lower ZnR/GPR39 mRNA level (inset, Fig. 2B) and completely lost the Zn^2+^-dependent Ca^2+^ response that was preserved in cells transfected with siControl (Fig. 2B,D). Silencing of GPR39 did not affect the response to ATP, indicating that ZnR/GPR39 is required for mediating Zn^2+^-dependent Ca^2+^ response in TAMR cells. Desensitization following prolonged exposure to the ligand is a hallmark of GPCRs activity, which is particularly prominent in ZnR/GPR39^37,39^. To determine whether Zn^2+^ induces desensitization of ZnR/GPR39, TAMR cells were incubated with Zn^2+^ for a prolonged period, using a lower concentration to avoid toxicity (100 μM for 25 min). Cells that were pre-incubated with Zn^2+^ exhibited lower response to Zn^2+^ compared to the response in control cells that were maintained in Ringer’s solution for the same time (Fig. 2C,D). To assess the recovery of the receptor, cells were returned to growth medium and imaged 24 hours later. A slightly increased Ca^2+^ response was triggered by Zn^2+^ (200 μM) in the cells that underwent desensitization (Fig. 2C,D). This suggests that the prolonged exposure to Zn^2+^ induces profound ZnR/GPR39 activation, likely depleting the intracellular Ca^2+^ stores in addition to the desensitization.

Transfection of MCF-7 cells with mCherry-tagged ZnR/GPR39 was followed by cell surface expression of the tagged receptor, but the mCherry-tagged vector (control) was accumulated within the cytoplasm of MCF-7 cells (Fig. 2E). The ZnR/GPR39 expressing MCF-7 cells exhibited Zn^2+^-dependent Ca^2+^ responses, monitored with Fura-2 (Fig. 2F,G). Finally, ZnR/GPR39 desensitization of the response was also monitored in MCF-7 mCherry-tagged ZnR/GPR39 cells that were pre-incubated with Zn^2+^ (Fig. 2G). Altogether, overexpression of ZnR/GPR39 in MCF-7 cells or induction of its expression in TAMR cells induces Zn^2+^-dependent Ca^2+^ signaling.

**Figure 3:**
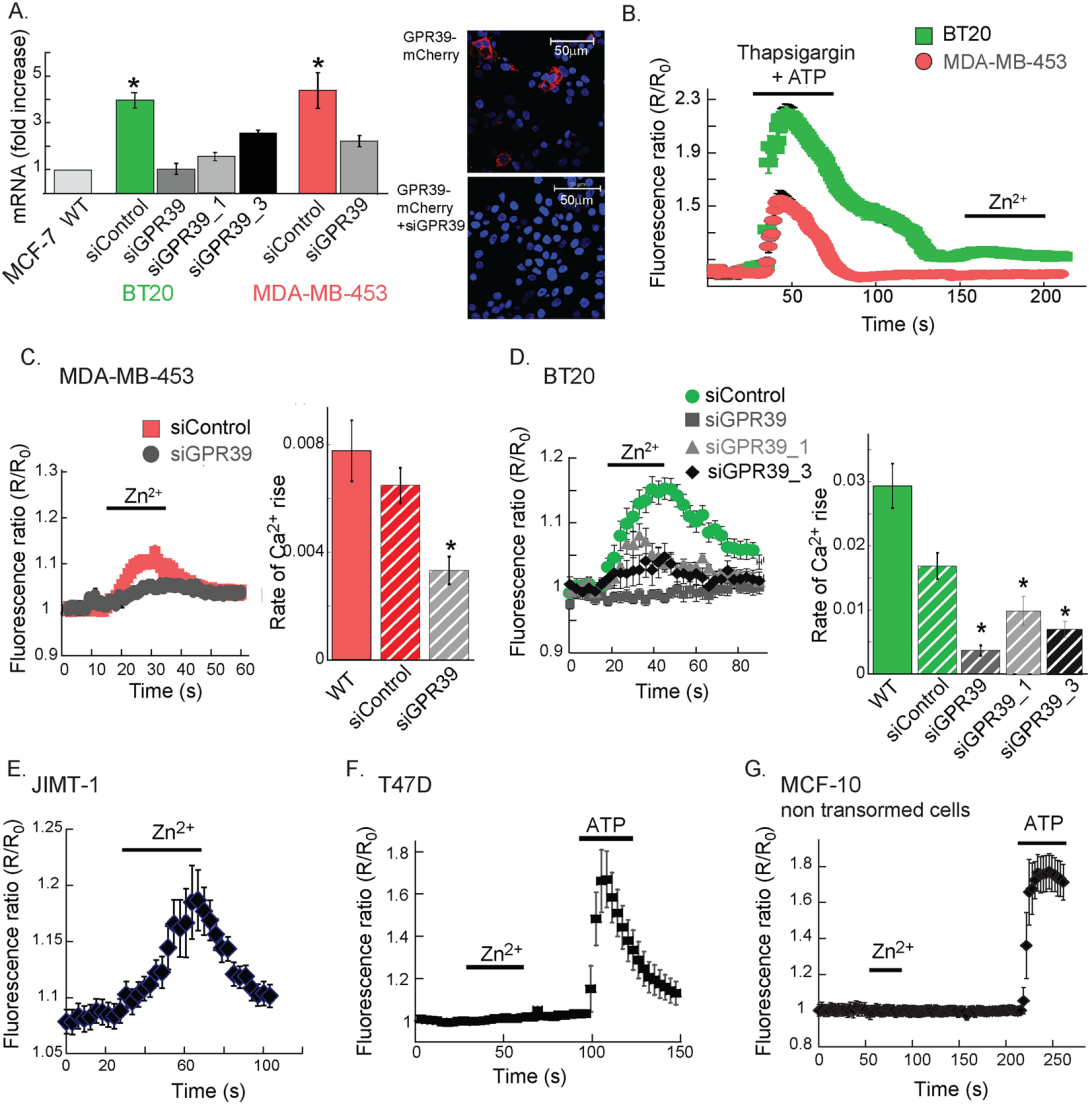
ZnR/GPR39 signaling is found in estrogen independent cell lines. (**A**) The mRNA level of GPR39 was determined using q-PCR in MCF-7, BT20 and MDA-MB-453 breast cancer cells, following transfection with siGPR39 constructs or scrambled (siControl) constructs (n = 3 for each cell line, p < 0.05 t-test). Right panel shows HEK293 cells transfected with mCherry-tagged GPR39 with siSCR or siGPR39 co-transfection, indicating that the siGPR39 efficiently attenuates ZnR/GPR39 expression. Representative confocal images showing Dapi (blue) nuclear staining and mCherry (red) indicating ZnR/GPR39 expression, which was abolished by siGPR39. (**B**) Fura-2 fluorescence was monitored in BT20 and MDA-MB-453 cells following Ca^2+^ store depletion (using 200 nM thapsigargin and 25 µM ATP, as in Fig. 1C), and application of Zn^2+^ (200 µM). (**C**) Representative Ca^2+^ response from siGPR39 or siControl MDA-MB-453 cells loaded with Fura-2 following application of Zn^2+^ (200 μM, at the indicated time). Average initial rates of the Ca^2+^ responses are shown in the right panel (n = 4 coverslips for each condition, in 3 independent experiments, p < 0.05 ANOVA). (**D**) Representative Ca^2+^ response to application of Zn^2+^ (200 μM, at the indicated time) in BT20 cells, loaded with Fura-2, transfected with several siGPR39 or siControl constructs. Average initial rates of the Ca^2+^ responses are shown in the right panel (n = 4 coverslips for each condition, in 3 independent experiments for WT, siGPR39 and siControl and 2 independent experiments for siGPR39_1 and siGPR39_2, p < 0.05 ANOVA). (**E**–**G**). Representative Ca^2+^ responses obtained as in C. using JIMT-1 (**E**); T47D (**F**); or MCF-10A (**G**) cells loaded with Fura-2 and treated with Zn^2+^ (200 μM) and ATP (25 µM), as indicated. (n = 3 coverslips for each condition, in 2 independent experiments).

To determine if Zn^2+^-dependent signaling is generally mediated in breast cancer cells, we studied ZnR/GPR39 activity in BT20, MDA-MB-453 cell lines, which are ERα and PR negative, but overexpress some level of HER2 (CCLE microarray data; https://portals.broadinstitute.org/ccle)^45^. In both BT20 and MDA-MB-453 cell lines, GPR39 mRNA levels were significantly higher than in MCF-7 cells, and its levels were reduced by siGPR39 (Fig. 3A). The siGPR39 construct also reduced expression of the mCherry-tagged GPR39 (inset). To verify that Zn^2+^ permeation is not induced in these cells, intracellular Ca^2+^ stores were depleted, using thapsigargin and ATP, and subsequent addition of Zn^2+^ did not trigger a fluorescent signal rise (Fig. 3B). We then asked if ZnR/GPR39 is active in these cells by monitoring the Zn^2+^-dependent Ca^2+^ release in Fura-2 loaded cells. Extracellular Zn^2+^ triggered Ca^2+^ responses in MDA-MB-453 (Fig. 3C) and BT20 (Fig. 3D), ER negative cells, and this Zn^2+^-dependent Ca^2+^ signaling was abolished in cells transfected with siGPR39 constructs (Fig. 3C,D). Furthermore, the ERα negative JIMT-1 cells also exhibited Zn^2+^-dependent Ca^2+^ responses (Fig. 3E), but the ERα positive T47D cells did not show Zn^2+^-dependent signaling (Fig. 3F), although both cell lines show high levels of GPR39 mRNA (CCLE microarry data; https://portals.broadinstitute.org/ccle). Importantly, non-transformed MCF-10A cells did not exhibit Zn^2+^-dependent Ca^2+^ responses (Fig. 3G). As a control, ATP induced robust Ca^2+^ responses in T47D and MCF-10A cells, indicating that the purinergic pathway activated by ATP is intact. Our results suggest a role for ZnR/GPR39 in mediating Ca^2+^ signaling in ER negative breast cancer cells, but not in cells that are ER positive.

**Figure 4:**
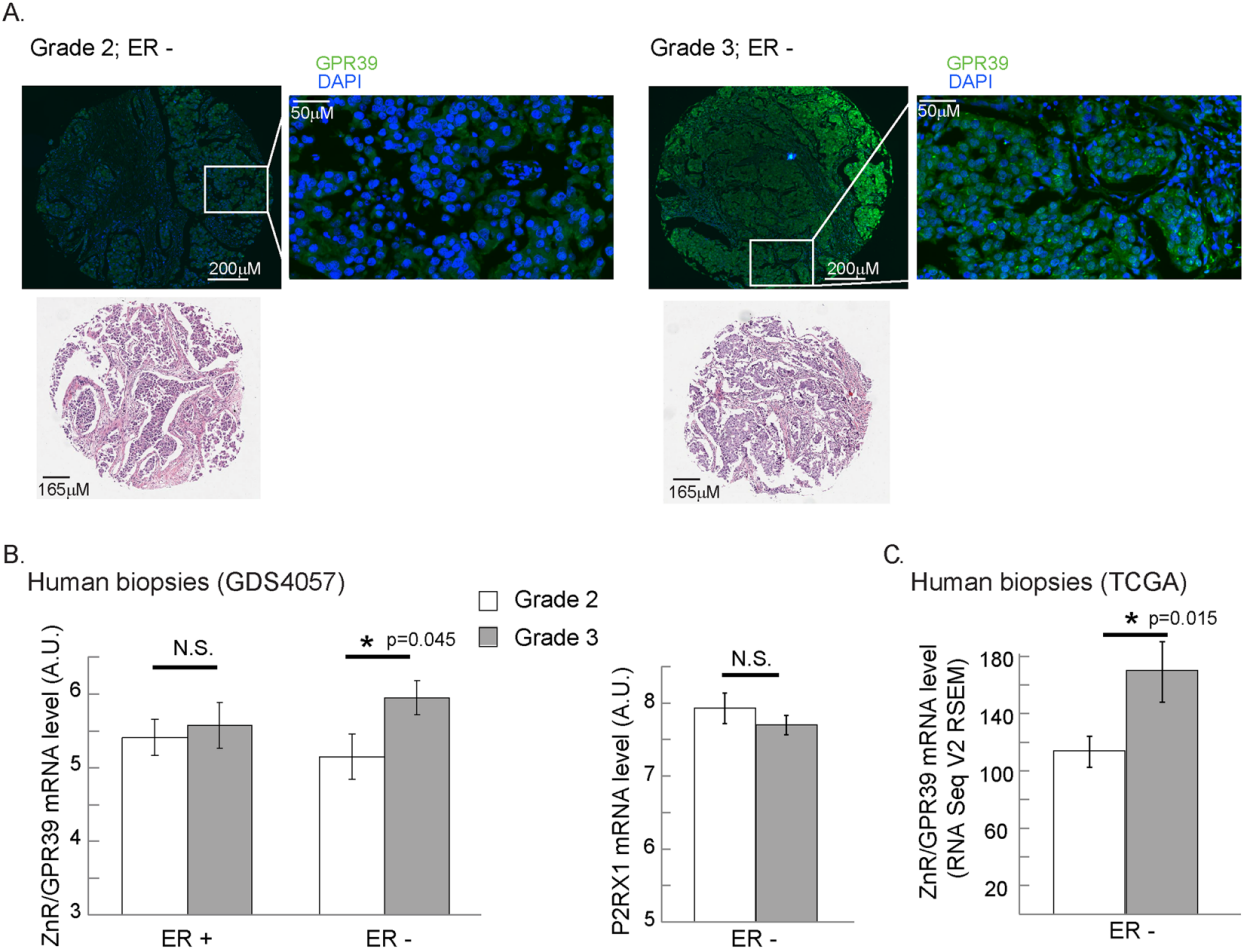
ZnR/GPR39 expression is increased in higher grade breast cancer tissue. (**A**) An array of breast cancer biopsies (http://www.biomax.us/tissue-arrays/Breast/BR1504a) was stained for ZnR/GPR39. Average staining level, above a threshold, in ER negative samples of grade 2 and grade 3 tissues is shown (scale bar shown on image). Representative images of ZnR/GPR39 staining and hematoxylin eosin stain (from the company site) are shown (bottom panel). (**B**) Analysis of ZnR/GPR39 mRNA level in ER-positive or negative, HER2-normal, breast cancer grade 2 (white) or 3 (grey) tumors (GDS4057 cohort, left panel). Note that a significant increase is seen in ZnR/GPR39 expression in ER-negative grade 3 tumors (p < 0.05 t-test). Right panel shows the analysis of mRNA level of P2XR1 purinergic receptor on the same cohort. (**C**) Analysis of ZnR/GPR39 mRNA level in ER-positive or negative, breast cancer stage 2 (white) or 3 (grey) tumors (TCGA cohort, p < 0.05, Welch’s t-test).

We then asked whether ZnR/GPR39 expression is associated with human breast cancer malignancy. We initially monitored the expression level of ZnR/GPR39 by immunostaining of breast cancer biopsies (http://www.biomax.us/tissue-arrays/Breast/BR1504a). Higher ZnR/GPR39 expression is seen in grade 3 biopsies compared to grade 2 (Fig. 4A, p = 0.04 by Fisher’s exact test) only in the ER-negative tissues, but not in the ER-positive tissues. Due to the small number of samples available in the tissue slide, we performed analysis for GPR39 expression using two independent gene expression datasets. In the HER2-normal breast cancer patients cohort, GDS4057^46^, gene profiling analysis was determined on pre-treatment biopsies, thus representing the initial expression level of the proteins. The dataset includes 103 ER-positive and ER-negative subtypes of breast cancer tissue mostly of grade 2 or grade 3. Analysis of all grade 3 versus grade 2 tissues indicated that there is a tendency towards increased GPR39 expression that did not reach statistical significance (p = 0.062). Interestingly, ER-positive tissues showed similar GPR39 expression in both grades, but the expression level of GPR39 in grade 3 ER negative tissues was significantly higher than in grade 2 ER-negative tissues (left panel Fig. 4B, p = 0.04). Importantly, expression of another Gq coupled receptor, the purinergic P2XR1, did not show a grade-dependent change (p = 0.3 for ER negative samples between grade 2 and grade 3, right panel Fig. 4B). To extend this mRNA expression analysis, we also explored an independent cohort of cancer patients, and compared the levels of GRP39 mRNA (RNA seq V2 RSEM) in breast cancer patients with stage 2 and stage 3 from the TCGA (www.cbioportal.org)^47–49^. Specifically, analysis of 197 ER-negative breast cancer patients showed that GPR39 was significantly higher in stage 3 compared to stage 2 (p = 0.022 Welch’s t-test, Fig. 4C). Levels of GPR39 mRNA expression were not significantly different in ER-positive biopsies from this cohort. These results suggest that increased expression of GPR39 is linked to breast cancer aggressiveness, particularly in ER-negative tumors.

### ZnR/GPR39 activates kinase signaling in breast cancer cells

**Figure 5:**
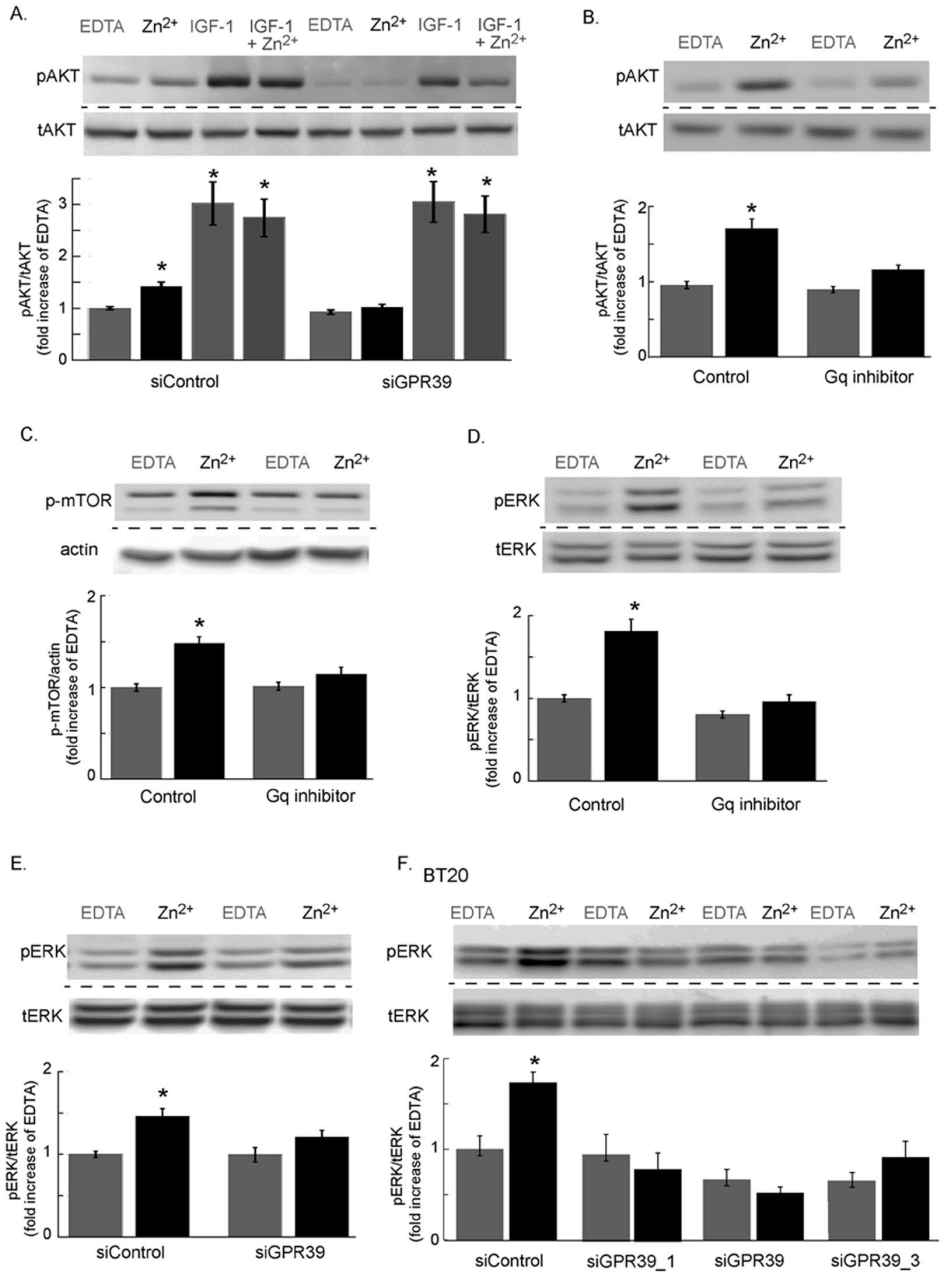
Activation of PI3K and MAPK pathways by ZnR/GPR39 in estrogen independent breast cancer cells. (**A**) Immuno-blots of phospho and total AKT in TAMR cells transfected with siGPR39 or siControl and treated with Zn^2+^ (200 μM) or IGF-1 (100 nM) or a combination of Zn^2+^ and IGF-1 or EDTA (100 μM) as control (top panel). Bottom panel shows densitometry analysis. (**B**) Immunoblot and denistometry analysis of pAKT and tAKT, as in A, of TAMR cells treated with or without the Gαq inhibitor, YM-254890 (1 μM, 30 min) and following Zn^2+^ (200 μM) application. (**C**) Immunoblot and denistometry analysis of phospho-mTOR levels in TAMR cells treated with or without the Gαq inhibitor, YM-254890 (1 μM, 30 min) and following Zn^2+^ (200 μM) application. (**D**) Immunoblot and densitometry analysis of pERK1/2, relative to total ERK1/2 in TAMR cells treated with or without the Gαq inhibitor, YM-254890 (1 μM, 30 min) and following Zn^2+^ (200 μM) application. (**E**) Immunoblot and denistometry analysis of pERK1/2, relative to total ERK1/2 in TAMR cells transfected with siGPR39 or siControl and treated with or without Zn^2+^ (200 μM). (**F**) Immunoblot and denistometry analysis of pERK1/2, relative to total ERK1/2 in BT20 cells transfected with several siGPR39 constructs or siControl and treated with or without Zn^2+^ (200 μM) (n = 2 independent experiments). Densitometry quantification in the bar graphs are averages of at least n = 3 independent experiments performed in triplicates for each condition (p < 0.05 ANOVA). Each blot presented was cropped from one original blot at one exposure. Original, full blots are presented in supplementary material.

We then asked if the enhanced activity of PI3K/AKT and MAPK/ERK1/2 pathways in breast cancer cells can be mediated via ZnR/GPR39. To specifically assess the role of ZnR/GPR39, we silenced ZnR/GPR39 expression in TAMR cells and monitored AKT and ERK1/2 phosphorylation, following a brief application of Zn^2+^ (200 μM, 30 s). An increase in phosphorylated AKT (pAKT) was observed in siControl cells, but not in siGPR39 transfected cells, that were treated with Zn^2+^ (Fig. 5A). To determine if a synergistic crosstalk between ZnR/GPR39 and IGF-1R can enhance PI3K/AKT activation, we also applied recombinant IGF-1 with or without Zn^2+^. While pAKT levels were higher when IGF-1 was applied, compared to Zn^2+^, co-application of both ligands did not further increase pAKT expression. Importantly, silencing of ZnR/GPR39 did not inhibit pAKT upregulation by IGF-1, although it abolished the Zn^2+^-dependent activation. Thus, Zn^2+^ via ZnR/GPR39 activated the PI3K/AKT independent of the IGF-1R pathway. When Zn^2+^ was applied in the presence of the Gαq inhibitor, YM-254890^38^, the Zn^2+^-dependent increase in pAKT was abolished (Fig. 5B), suggesting that ZnR/GPR39 signaling induces the phosphorylation. Increased phosphorylation of mTOR, which is downstream to the PI3K, was also monitored following treatment with Zn^2+^ (200 μM) in control TAMR cells, but not in cells treated with Gαq inhibitor (Fig. 5C). Similarly, Zn^2+^-induced increase in phosphorylated ERK1/2 (pERK1/2) that was reversed by the Gαq inhibitor (Fig. 5D) and by transfection with the siGPR39 construct (Fig. 5E). Finally, pERK1/2 was also increased in BT20 cells transfected with siControl but not in cells transfected with siGPR39 constructs (Fig. 5F), which attenuated the Zn^2+^-dependent Ca^2+^ response (Fig. 3F). Thus, Zn^2+^ and ZnR/GPR39, via a Gαq-dependent pathway, upregulate the PI3K/AKT and MAPK pathways in TAMR and BT20 breast cancer cells.

### ZnR/GPR39 enhances proliferation and invasiveness of breast cancer cells

**Figure 6:**
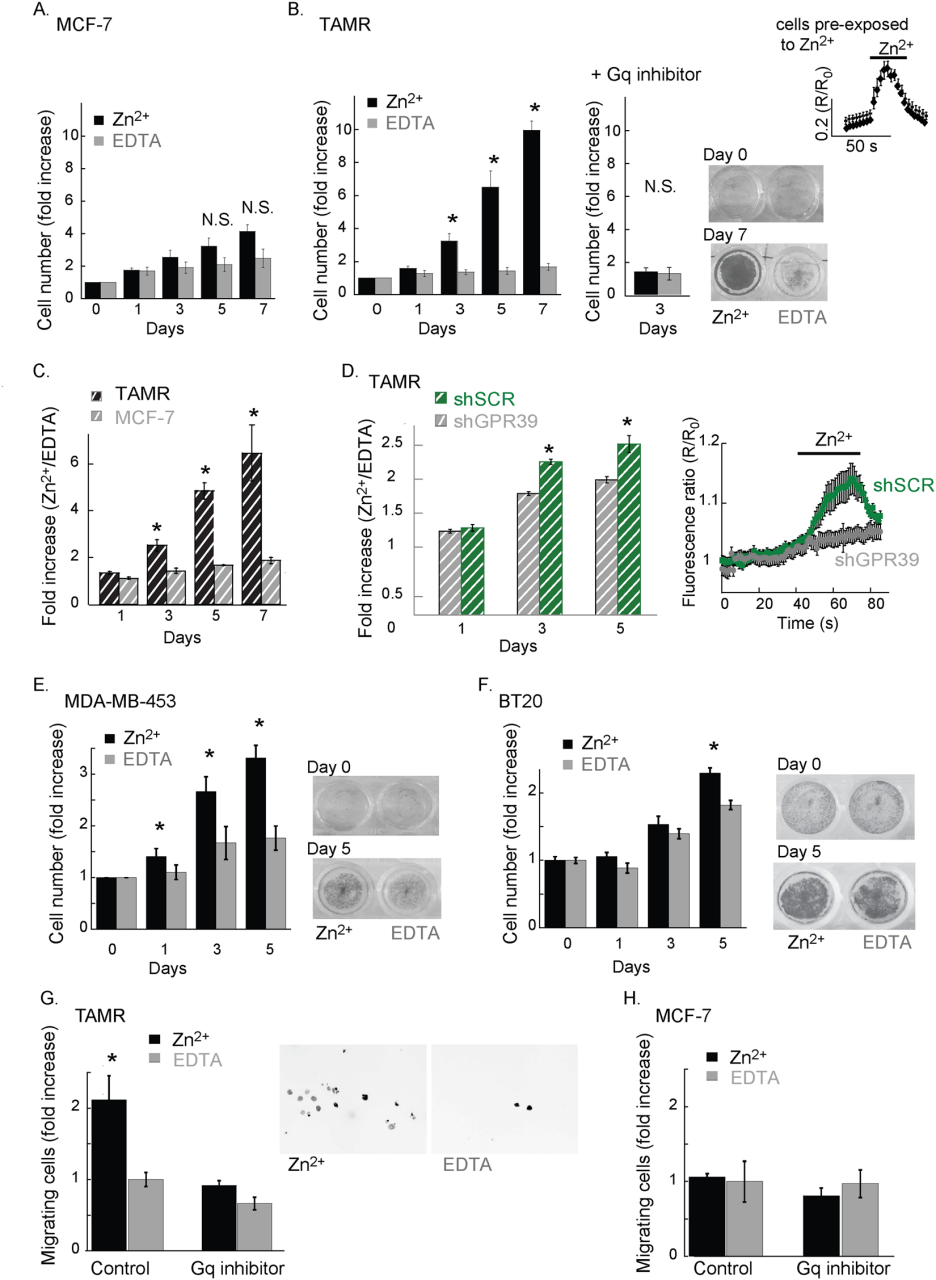
Cell growth and invasion are increased by ZnR/GPR39. Cell growth, as measured using SRB levels, in cells treated daily with Zn^2+^ (200 μM, 2 min) to activate ZnR/GPR39 versus EDTA (100 μM, 2 min) used to chelate residual or released Zn^2+^. (**A**) Averaged count of numbers of MCF-7 cells following Zn^2+^ or EDTA daily treatment (p < 0.05 t-test, between treatments for each day). (**B**) Left panel shows averaged TAMR cell numbers, as in A. (p < 0.05 t-test, between treatments for each day). Middle panel shows the cell numbers in cells pre-treated with the Gαq inhibitor (YM-254890, 10 μM, 30 min) prior to the daily treatment with Zn^2+^ (200 μM, 2 min. n = 5). Bottom right panel shows representative images of the plate following SRB staining on day 0 (no Zn^2+^ treatment) versus day 7 of treatment. Top right panel shows representative Ca^2+^ responses monitored using Fura-2 fluorescence as in Fig. 1, in TAMR cells that were pre-treated with Zn^2+^ (200 μM, 2 min) 24 hrs. prior the imaging (n = 4 coverslips). (**C**) Analysis of A-B showing the daily fold increase in cell number, as monitored with SRB, following Zn^2+^ treatment compared to EDTA treatment (p < 0.05 t-test, between daily treatment of MCF-7 and TAMR cells). (**D**) TAMR cells were infected with shGPR39 lentiviral constructs or shSCR (scambled control), as described. Cell growth analysis using SRB was done as in A, and number of cells treated with Zn^2+^ were normalized compared to cells treated with EDTA (as in C; p < 0.05 t-test, between daily treatment of shSCR and shGPR39 cells). Right panel shows representative Ca^2+^ responses monitored using Fura-2 fluorescence in the shGPR39 and shSCR cells, indicating silencing abolished the Zn^2+^-dependent Ca^2+^ signaling. (n = 2 independent experiments (p < 0.05 t-test, between shSCR and shGPR39 each day). (**E**) Averaged MDA-MB-453 cell numbers for Zn^2+^ or EDTA treatment on each day, the right panel shows representative images of the plate following SRB staining. (p < 0.05 t-test, between treatments) (**F**) Averaged BT20 cell numbers for Zn^2+^ or EDTA treatment on each day; the right panel shows representative images of the plate following SRB staining. (p < 0.05 t-test, between treatments) (**G**) Migration of cells via matrigel was determined, for TAMR cells treated with or without the Gαq inhibitor, YM-254890 (1 μM, 30 min). Cells were treated with Zn^2+^ (200 μM, 2 min) or EDTA (100 μM, 2 min) as control, and cells that migrated through Matrigel were counted (20×, DAPI staining, inversed greyscale image). The average numbers of cells are shown in the bar graph (left panel). Right panel shows a representative image of the cells that invaded through Matrigel at 72 h following treatment (Zn^2+^ or EDTA). (n = 3 independent experiments, p < 0.05 ANOVA). (**H**) Migration of MCF-7 cells using the paradigm described in F.

To determine if activation of ZnR/GPR39 signaling controls breast cancer cell growth and invasion, we initially compared the Zn^2+^-dependent cell growth rates in ZnR/GPR39-expressing TAMR cells, and MCF-7 cells deficient in this receptor. Cells were treated daily, for 7 consecutive days, with Zn^2+^ (200 μM, 2 min) or EDTA (100 μM, 2 min) a paradigm that does not induce desensitization of ZnR/GPR39 or Zn^2+^ permeation into the cells. To chelate extracellular Zn^2+^, which is a contaminant in many solutions or may be released endogenously from cells, we applied the cell impermeable chelator EDTA that is a high affinity Zn^2+^ chelator (Kd 10^−13^ M^50^), at a concentration that does not affect extracellular Ca^2+^, as previously done^37,39,40^. Application of extracellular Zn^2+^ did not significantly enhance proliferation of MCF-7 cells (Fig. 6A), but did enhance proliferation of TAMR cells by more than 3-fold already on the 3^rd^ day of treatment (Fig. 6B). Importantly, the short Zn^2+^ transients applied daily did not desensitize or attenuate ZnR/GPR39 Ca^2+^ responses (top right panel, 6B). In the TAMR cells that express ZnR/GPR39, cell growth was not enhanced by Zn^2+^ when applied in the presence of the Gαq inhibitor (YM-254890, 10 μM, 30 min, middle panel). To determine the effect of Zn^2+^ compared to EDTA, we analyzed the ratio between the numbers of cells treated with Zn^2+^ or EDTA on each day. We found that Zn^2+^-dependent cell growth in the TAMR, ZnR/GPR39-expressing, breast cancer cells was much faster compared to MCF-7 cells (Fig. 6C). We then determined the specific role of ZnR/GPR39 using shGPR39 silencing with lentiviral constructs, more efficient than the siGPR39 transfection. Infection of the cells with the lentiviral constructs abolished the Zn^2+^-dependent Ca^2+^ responses in shGPR39 TAMR cells, but not in the shSCR cells infected with a scrambled construct, suggesting that ZnR/GPR39 signaling is absent in the shGPR39 cells (Fig. 6D, right panel). Growth rates of TAMR cells infected with shSCR showed Zn^2+^-dependent enhancement of cell numbers compared to EDTA treated cells; but the effect of Zn^2+^ was significantly attenuated in the ZnR/GPR39 silenced (shGPR39) cells (Fig. 6D). We also compared proliferation rates of ZnR/GPR39 expressing MDA-MB-453 and BT20 breast cancer cells following daily treatment with Zn^2+^ (200 μM, 2 min) or EDTA (100 μM, 2 min). In both ZnR/GPR39-expressing cell lines, Zn^2+^ treatment significantly increased the number of cells compared to EDTA (Fig. 6E,F). While the Zn^2+^ increased cell growth was clearly seen in the MDA-MB-453 cells already following one daily treatment (Fig. 6G), the BT20 cells presented very slow growth rates and Zn^2+^ slightly, albeit significantly, enhanced BT20 cell growth only the 5^th^ day of treatment (Fig. 6F). These results suggest that Zn^2+^ enhances cell growth in ZnR/GPR39-expressing breast cancer cells.

We next examined if Zn^2+^ controls the invasiveness of TAMR or MCF-7 cells by monitoring their growth into a matrigel matrix following daily Zn^2+^ or EDTA treatments (same paradigm described above). To specifically determine the role of ZnR/GPR39, Zn^2+^ was applied in the presence or absence of the Gαq inhibitor. TAMR cells that were treated with Zn^2+^ showed about two-fold higher invading cell numbers, and this effect was reversed by application of the Gαq inhibitor (Fig. 6G). In contrast, Zn^2+^ did not enhance MCF-7 invasiveness compared to control cells, nor was this affected by the Gαq inhibitor (Fig. 6H). Therefore, we suggest that Zn^2+^ enhances invasiveness of TAMR cells, but not of MCF-7 cells, via the Gαq signaling triggered by ZnR/GPR39.

## Discussion

The tamoxifen-resistant TAMR cells exhibited cellular Zn^2+^ dyshomeostasis and increased CK2 phosphorylation^22,23^ that was linked to a more aggressive phenotype of these cells^25^. A role for ZnR/GPR39 in enhancing signaling and cell growth was not addressed. We show that ZnR/GPR39 expression and function are increased in TAMR cells, compared to the original MCF-7 cells. Importantly, changes in intracellular Zn^2+^ do not induce direct ZnR/GPR39 signaling^33,35^, but the higher levels of accumulated Zn^2+^^20,51^ may suggest that transient extracellular changes by release of Zn^2+^^39^ can trigger ZnR/GPR39 activation and enhanced cell growth. Importantly, ZnR/GPR39 is essential for Zn^2+^-dependent signaling leading to enhanced cell growth in the TAMR cells as well as in MDA-MB-453 cells and BT20 cells. Cell signaling and subsequent increases in proliferation rate were triggered by transient extracellular Zn^2+^ treatment in ERα-negative breast cancer cells expressing a functional ZnR/GPR39. Thus, we suggest that changes in Zn^2+^ homeostasis in breast cancer tissue, together with increased ZnR/GPR39 expression yield an alternative pathway leading to cell growth.

The affinity of ZnR/GPR39 is modified in various tissues, such that in colonocytes the apparent Km was 80 ± 15 µM, but in keratinocytes Km was two orders of magnitude higher, 450 ± 50pM. Both concentrations are physiologically relevant in the relative tissue^34,36,52^. The different affinities of ZnR/GPR39 may be regulated by its interaction with other GPCRs as is common in this family of receptors^53^. Alternatively, the presence of other ions may modulate ZnR/GPR39 affinity to Zn^2+^^38,52^. Interestingly, the Ca^2+^ sensing receptor, which interacts with ZnR/GPR39^38^, has been shown to promote breast cancer^54^. The differences in the apparent affinity and maximal activity of ZnR/GPR39 in TAMR versus MCF-7 cells may also result from differences in mRNA levels. Importantly, the concentration of “elemental zinc” in breast cancer tissue is estimated to be of about 100 µM^55^, as measured by X-ray fluorescence. This measurement does not distinguish between the localization (extra or intracellular or organellar) or the binding of ionic Zn^2+^ to proteins. Nevertheless, and in agreement with reports describing changes in vesicular Zn^2+^ as well as “elemental zinc” in breast cancer cells, potential Zn^2+^ release from protein or vesicles may be sufficient for triggering ZnR/GPR39 activation. The release of Zn^2+^ induces transient changes in extracellular Zn^2+^ because this ion undergoes re-uptake into the cells via ZIP transporters or is chelated by binding to proteins^15,56^. The increase in ZnR/GPR39 level in more aggresive cancer cells taken together with such transient Zn^2+^ changes may therefore play a role in enhanced growth and invasiveness.

Analysis of human breast cancer tissues, using the GDS4057 cohort^46^ or the TCGA^49^ available data, indicated that in ERα-negative tissues increased ZnR/GPR39 expression is linked to more aggressive phenotypes. Importantly, in ERα-positive biopsies from both cohorts the levels of ZnR/GPR39 expression was not significantly different between phenotypes. In addition, using human breast cancer tissue staining, we also show an increase in ZnR/GPR39 expression in grade 3 tissues compared to grade 2. Changes in the expression of ZnR/GPR39 were previously associated with more aggresive cancers. For example, GPR39 was suggested as a biomarker for ovarian cancer since mRNA level is 6-fold higher in ovarian cancer tissue samples compared with normal tissue^57^. Similar increase in GPR39 expression was found in human esophageal squamous cell carcinoma and was linked to enhanced cell growth in the KYSE30 esophageal carcinoma cell line^58^. While a direct role for Zn^2+^ was not determined, upregulation of GPR39 was also demonstrated in aggressive cancer cell lines of breast, ovarian and prostate, and melanoma^59^ and was linked to EGFR transactivation^60^. Moreover, GPR39 overexpression in porcine intramuscular preadipocytes enhanced their differentiation and proliferation via the PI3K pathway. Finally, GPR30 is an estrogen-specific orphan GPCR^61^ that triggers cAMP release in SKBR3 breast cancer cell line^62^. GPR30 also mediates proliferative effects, induced by 17β-estradiol and hydroxytamoxifen, in endometrial cancer cells^63^. Our results indicate that ZnR/GPR39 signaling, triggered by extracellular Zn^2+^, leads to activation of the MAPK and PI3K/AKT pathways, both closely associated with cell growth and survival in cancer. We further show that ERα-negative TAMR, BT20 and MDA-MB-453 cells, exhibit increased proliferation rates following ZnR/GPR39 activation, compared to the ERα-positive MCF-7 cells that do not have ZnR/GPR39 signaling. Moreover, invasion of TAMR cells following ZnR/GPR39 activation was increased, and this was abolished by the Gq inhibitor or in the MCF-7 cells, suggesting that ZnR/GPR39 signaling is essential for Zn^2+^-dependent increased migration. The data presented here support our hypothesis that ZnR/GPR39 mediates signaling pathways leading to aggressive growth in breast cancer cells, particularly in the absence of the estrogen receptor pathway. Thus, ZnR/GPR39 may provide an alternative pathway that contributes to increased malignancy in breast cancer.

Activation of the PI3K/AKT pathway in breast cancer cells is not only enhancing cell survival but was also associated with endocrine resistance^8^. Activation of an upstream receptor such as the IGF-1 receptor is associated with the initial upregulation of this pathway^64^. Our results show that Zn^2+^ can similarly activate the PI3K pathway via ZnR/GPR39 and, that this is independent of the IGF-1 pathway. Activation of both AKT and mTOR, as well as the MAPK pathway, by Zn^2+^, were mediated by ZnR/GPR39-dependent activation of a Gαq pathway. This pathway may also lead to phosphorylation of ZIP7 that induces subsequent Zn^2+^ release and prolonged activation of PI3K and MAPK^22^. Both, the MAPK and PI3K pathways, play a prominent role in breast cancer tumorigenesis^8,65^ and inhibitors of these pathways have been suggested for use in addition to the antihormone therapy. However, direct inhibition of molecules in the PI3K or MAPK pathways leads to activation of negative feedback loops which further enhance cell growth^66–69^. Our study suggests that in breast cancer tissue, elevated Zn^2+^ levels and increased ZnR/GPR39 expression may provide an alternative pathway to PI3K and MAPK activation. Activation of ZnR/GPR39 by endogenous Zn^2+^ in breast cancer tissue may occur following specific release of Zn^2+^ from vesicles^16,31,51^ or cell injury and death^39^.

Altogether the results presented support an important role for ZnR/GPR39 in mediating Zn^2+^-signaling that is leading to enhanced growth and invasiveness of breast cancer cells. Changes in the expression of ZnR/GPR39 in breast cancer cells following loss of the ER signaling may provide a circumventing pathway for metabotropic signaling activation and enhanced cell growth in breast cancer. As such, ZnR/GPR39 may provide an upstream target for novel therapeutic approaches for hormone resistant breast cancer.

## Methods

### Cell culture

MCF-7 cells were grown in standard RPMI medium (Gibco, Scotland) supplemented with 5% (v/v) fetal calf serum (Biological Industries, Israel) and 1% pen-strep solution (10000 U/ml penicillin, 10000 µg/ml streptomycin, Sigma-Aldrich, Israel) and 1% L-Glutamine. TAMR cells (a gift from JMW Gee, Cardiff University) derived from MCF-7 cells that were grown for 6 months in tamoxifen antihormone treatment^70^, were grown in RPMI medium without phenol red (Gibco, Scotland), supplemented with 5% (v/v) charcoal stripped, steroid-free, fetal calf serum (Biological Industries, Israel), 1% pen-strep solution, 2% L-Glutamine (200 mM) and 4-hydroxytamoxifen (4-OHTAM, 0.1 µM in ethanol, Sigma-Aldrich, Israel). For comparison between the cell types, MCF-7 cells were grown in the same medium as TAMR cells, but without tamoxifen. MDA-MB-453 and T47D cells were grown in standard RPMI medium (Gibco, Scotland), supplemented with 10% (v/v) fetal calf serum (Biological Industries, Israel) and 1% pen-strep solution. BT20 cells were grown in Eagle’s Minimum Essential Medium (EMEM, Biological Industries, Israel) supplemented with 10% (v/v) fetal calf serum (Biological Industries, Israel) and 1% pen-strep solution. All cells were grown in a 5% CO_2_ humidified atmosphere at 37 °C. JIMT-1 cells were grown in Dulbecco modified Eagle medium (DMEM, Biological Industries, Israel) supplemented with 10% (v/v) fetal calf serum (Biological Industries, Israel), 1% pen-strep solution (10000 U/ml penicillin, 10000 µg/ml streptomycin, Sigma-Aldrich, Israel) and 1% L-Glutamine. MCF-10A cells were grown in DMEM/F12 (Gibco, Scotland) supplemented with 10% (v/v) fetal calf serum (Biological Industries, Israel), 1% pen-strep solution (10000 U/ml penicillin, 10000 µg/ml streptomycin, Sigma-Aldrich, Israel) 1% L-Glutamine, EGF (20 ng/ml), Hydrocortisone (0.5 mg/ml) and human Insulin (10 µg/ml).

### Cell transfection and infection

For GPR39 silencing, TAMR, MCF-7, MDA-MB-453 and BT20, cells were seeded in 60 mm plates, 24 h prior to transfection in media containing serum but without antibiotics. Cells were transfected with siRNA using Lipofectamine 2000 (Thermo-Fisher Scientific, MA, USA) according to the manufacturer’s protocol. The siRNA constructs used for GPR39 silencing: 5′-CCAUGGAGUUCUACAG CAU-3′ (siGPR39, Sigma-Aldrich, Israel), 5′-CUCCCAAAUCAUUGAUCACAGUCAT-3′ (siGPR39_1, Integrated Data Technologies, Belgium), 5′-GCAUCAUCUGGAAUCCCCUGACCAC-3′ (siGPR39_3, Integrated Data Technologies, Belgium), and siRNA control (scrambled) was 5′- GCCCAGAUCCCUGUACGU-3′. Cells were used for experiments 48 h after transfection. For GPR39 overexpression, MCF-7 cells were seeded in 60 mm plates, 24 h prior to transfection, in RPMI phenol-free media containing serum but without antibiotics. Cells were transfected with a plasmid carrying GPR39 with m-Cherry sequence or m-Cherry vector alone, as control. Transfections were done with TransIT-BrCa (Mirus Bio, WI, USA), according to the manufacturer’s protocol. Experiments were performed 48 h following transfection. TAMR cells were infected using MISSION Lentiviral Transduction Particles (Sigma-Aldrich) according to manufacturer protocol. Briefly, 50,000 cells were seeded a day before infection on 24-well plates. Infection was carried out using five lentivirus clones carrying DNA sequences coding for shRNA compatible with hGPR39 (shGPR39_1:CCGGCCTCCAATATGTCCATCTGTACTCGAGTA CAGATGGACATATTGGAGGTTTTT; shGPR39_2:CCGGGAACATGATGCAGGTGCTC ATCTCGAGATGAGCACCTGCATCATGTTCTTTTT; shGPR39_3:CCGGGCTGCAGAAGA AAGGATACTTCTCGAGAAGTATCCTTTCTTCTGCAGCTTTTT; shGPR39_4:CCGGG TACCTGATCATCTTCGTGATCTCGAGATCACGAAGATGATCAGGTACTTTTT; shGPR39_5:CCGGGAACATGATGCAGGTGCTCATCTCGAGATGAGCACCTGCATCATGTTCTTTTT, or scrambled sequences. Twelve hours post infection the medium was replaced with growth medium.

### Fluorescent Imaging

The imaging system consisted of an Axiovert 100 inverted microscope using a 20× objective (Zeiss, Germany), Polychrome V monochromator (TILL Photonics, Germany) and a SensiCam cooled charge-coupled device (PCO, Germany). Fluorescent imaging measurements were acquired with Imaging Workbench 5 (Indec, USA). All results shown are the means of at least three independent experiments, with averaged responses of 20 cells in each experiment. Bar graphs show the initial rates of fluorescence change averaged over at least n slides from 3 independent experiments, as mentioned in figure legends.

### For Ca^2+^ imaging

cells were seeded on coverslips at least 24 h prior to the experiment, and loaded with Fura-2 acetoxymethyl ester (AM; 25 min 2 μM; TEFLabs Inc., TX, USA) in Ringer’s solution with 0.1% BSA (Bovine Serum Albumin, Sigma-Aldrich, Israel), and subsequently washed for at least 15 min. Coverslips were then mounted in a microscope chamber that allowed rapid solution exchange. Fura-2 was excited at 340 nm and 380 nm and imaged with a 510 nm long-pass filter. Activation of ZnR/GPR39 was triggered by application of 200 µM Zn^2+^ in Ca^2+^ free Ringer’s solution^71,72^ for 30 s up to 2 min, as indicated in figures, a paradigm that did not induce cytoplasmic Zn^2+^ rise (see Fig. 1C,D). The initial rate of the fluorescent signal rise, calculated over 20 s, was used to determine the response.

### For Zn^2+^ imaging

cells were seeded as described and loaded with Fluozin-3 (AM; 30 min 25 μM; Invitrogen, CA, USA) in Ringer’s solution with 0.1% BSA and subsequently washed for at least 25 min. The excitation was at 480 nm, and emission was monitored through a 510 nm long- pass filter. The cells were incubated for 2 or 20 minutes with 100 µM Zn^2+^ and the cell permeable Zn^2+^ chelator TPEN (N,N,N′,N′-tetrakis (2-pyridylmethyl) ethylenediamine, 10 μM, Sigma-Aldrich, Israel) was added while monitoring cellular fluorescence. Image acquisition was done at 20× using an Olympus BX51 fluorescent microscope with a CCD. Quantitative analysis was performed by counting pixels that were stained above a predetermined threshold, normalized to the number of cells in the slide and compared to control values.

### Western blots

Protein expression was monitored using western blot analysis. Cells were treated with Zn^2+^ or control (as described) and harvested into lysis buffer (0.5% Deoxycholic acid, 25 mM NaF, 10 mM Na_3_PO_4_, 1 mM sodium-orthovanadate, 100 mM NaCl, 5 mM EDTA, 5 mM EGTA, 2% (v/v) Triton X-100) in the presence of Protease Inhibitor Cocktail (1:50 Complete, Sigma-Aldrich, Israel). For testing phosphorylation of ERK1/2 or AKT the buffer also included PNPP (20 mM, Sigma-Aldrich, Israel), sodium vanadate (1 mM) and NaF (25 mM). Lysates were placed on ice for 10 min and then centrifuged for 30 min (12,000 rpm) at 4 °C. Supernatants (cytosolic fraction) were collected, protein concentrations were determined using Bio-Rad protein assay (Bio-rad, CA, USA), SDS sample buffer was added, and samples were boiled for 5 min and then frozen at −80 °C until used. Kinase phosphorylation was assayed using cell lysates (25 μg), separated on a SDS-PAGE (10% for ERK1/2, AKT and occludin or 7.5% for mTOR) and analyzed using antibodies raised against the doubly phosphorylated ERK1/2, total ERK1/2 and total AKT (Sigma-Aldrich, Israel) or phosphorylated AKT (Santa-Cruz Biotechnology, TX, USA) phosphorylated mTOR (1:500, Cell Signaling, MA, USA), actin (1:50,000,MP-Biomedicals, UK). Densitometry analysis of expression level was performed using EZQuant-Gel software (EZQuant, Israel). Phosphorylation of ERK1/2, AKT and mTOR were normalized to total ERK1/2, total AKT or actin respectively. For each experiment, activation by Zn^2+^ (200 µM) was normalized to the response triggered following treatment with EDTA (100 µM). The bar graphs represent the averaged response of at least three independent experiments performed in triplicates.

### For analysis of kinase activation

TAMR or MCF-7 cells, transfected with siRNA were serum starved for at least 4 h and then treated with Zn^2+^ (200 μM, 30 s) or recombinant IGF-1 (100 nM for 30 s, Abcam, UK) or EDTA (Ethylenediaminetetraacetic acid used as control at 100 μM, a concentration that does not affect Ca^2+^ but chelates any excess Zn^2+^, 30 s) in Ca^2+^-free Ringer’s solution. Cells were then incubated for in Ca^2+^ free Ringer’s solution (20 min for analysis of AKT or mTOR phosphorylation, or 5 min for ERK phosphorylation). In another set of experiments, TAMR and MCF-7 cells were incubated for 30 min with the Gq inhibitor YM-254890 (1 μM, Astella Pharma, Japan) and then treated as described. Levels of pERK1/2 and pAKT were normalized against the total ERK1/2 or AKT protein, respectively.

### Real-Time PCR

To determine GPR39 mRNA level, cells were seeded on 60-mm plates and scraped after 48 h using RNeasy Mini Kit lysis buffer (QIAGEN, Germany). Cell lysates were homogenized using QIAshredder and total RNA was purified using RNeasy Mini Kit as described by the manufacturer (QIAGEN, Germany). 1 μg RNA was converted to cDNA using qScript cDNA synthesis kit (Quanta Biosciences, MA, USA). 20 ng of the cDNA was subjected to real time PCR procedure (Taqman, Applied Biosystems, Thermo Scientific), which was done with ABsolute Blue QPCR kit as described by the manufacturer (Thermo Scientific). Primers and probes were supplied by (Solaris, Thermo Scientific) sequence for GPR39: forward primer CATCTTCCTGAGGCTGA, reverse primer ATGATCCTCCGTCTGGTTG, probe TATGCT GGATGCCCAAC, and for Actin: forward primer TGGAGAAAATCTGGCACCAC, reverse primer GGTCTCAAACATGATCTGG, probe ACCGCCAGAAGATGACC.

### Proliferation assay/SRB assay for cell density

Sulforhodamine B (SRB) assay was used to assess cell proliferation. TAMR and MCF-7 cells were seeded into 24-well plates (60,000 cells per well) and grown in starvation medium: phenol-free RPMI medium containing 1% serum, and 100 µM EDTA, to chelate basal Zn^2+^ levels, for 24 h. MDA-MB-453 cells were seeded into 24-well plates (80,000 cells per well) and supplied with RPMI starvation medium with 100 µM EDTA. BT20 cells were seeded into 24-well plates (30,000 cells per well) and supplied with EMEM starvation medium with 100 µM EDTA. For daily activation of ZnR/GPR39 activation, cells were transferred into Ca^2+^ free Ringer’s solution and treated with Zn^2+^ (200 µM, 2 min) or 100 µM EDTA (as control) and then returned to the starvation medium for another 24 h. This procedure was repeated during 5–7 consecutive days. Cells were fixed on days 0, 1, 3, 5 and 7 using 10% TCA (trichloroacetic acid) for 1 h at 4 °C. The supernatant was discarded and plates were washed with deionized water and air-dried, then 300 µl sulforhodamine B (SRB 0.4 w/v in 1% acetic acid) was added to each well (10 min at room temperature). The unbound SRB was removed by washing with 1% acetic acid and the plates were air-dried. The dye bound to basic amino acids of the cell membrane was solubilized with Tris buffer (10 mM, pH 10.5) and the absorption measured at 540 nm by ELISA reader (Molecular Devices, CA, USA). Cell number was quantified using a calibration curve, and cell proliferation was determined as percentage of the number of cells monitored on day 0 (non-treated, 100%). Each bar graph represents an average of at least three independent experiments.

### Invasion

For invasion experiments, growth factor-reduced invasion chambers (Corning) with 8.0 μm PET membrane, coated with Matrigel, were used. 50,000 TAMR or MCF-7 cells were seeded in each chamber. At 24 h and 48 h cells were treated for 2 min with Zn^2+^ (200 µM) or EDTA (100 µM), as control, in Ca^2+^ free Ringer’s solution, with or without the Gq inhibitor, YM-254890 (1 μM). After treatment the cells were given starvation medium, whereas the well itself contained the regular growth medium of each cell type. At 72 h, matrigel was removed using a cotton swab and cells were fixed with 4% PFA. The membranes were removed from the chamber using a scalpel blade, and mounted onto a glass microscope slide with a mounting solution containing DAPI (Fluoromount-G, Southern Biotech, Thermo Scientific). Cells were counted at 20× magnification in a blind manner. At least three different membranes were counted for each treatment.

### Immunofluorescence

To determine GPR39 expression in human breast tumors, a breast cancer tissue array was used (Biomax, USA cat # BR1504a). Tissues were de-paraffinized, and after heat-induced antigen retrieval (95 °C, 10 min) the tissues were blocked with normal goat serum for 20 min and incubated overnight at 4 °C with primary anti-rabbit GPR39 antibody (1:100, Abcam, UK Cat #: AB18859, see negative control in Supplementary data 1) and 1 h at room temperature with secondary rabbit Cy2-conjugated antibody (1:100, Jackson ImmunoResearch Laboratories, Inc., PA, USA). A cover slide was mounted using a solution containing DAPI. The slide was digitalized using the Pannoramic Scanner (3DHISTECH, Budapest, Hungary) and analyzed using QuantCenter (3DHISTECH, Budapest, Hungary) using a single threshold parameter for all images. Briefly, a person blind to the tissue data determined a threshold value in the stained biopsies, this value of 75 (scale of 0–254 used on the images) was used for the analysis of the whole slide. The percentage of pixels in each biopsy that had a value above this threshold was calculated and biopsies with any percentage above 0 were marked as high expression of GPR39. The numbers of positive GPR39- expressing biopsies versus negative expressing biopsies was determined, and the tissues were classified as grade 2 or 3 and ER negative or positive expression, as done previously^73^. We then performed a Fisher analysis for the slides that exhibited negative or positive ER, separately, and determined the significance of the difference between grade 2 and 3 that were with or without GPR39 expression.

### Statistical Analysis

Data are expressed as means ± SEM. Each treatment was compared with the control or Zn^2+^ treatment, and statistical significance between the groups was evaluated using the Student’s t-test or ANOVA, as marked in figure legends. *p < 0.05.

## Electronic supplementary material

Supplementary Information
