# Supplementary material for: Enhanced ZnR/GPR39 Activity in Breast Cancer, an Alternative Trigger of Signaling Leading to Cell Growth
Source: Sci Rep. 2018 May 25;8:8119. doi: 10.1038/s41598-018-26459-5 (PMC5970167; doi:10.1038/s41598-018-26459-5)

**Enhanced ZnR/GPR39 Activity in Breast Cancer, an Alternative Trigger of Signaling Leading to Cell Growth**

Hila Ventura-Bixenshpaner*, Hila Asraf, Moumita Chakraborty, Moshe Elkabets, Israel Sekler, Kathryn M. Taylor and Michal Hershfinkel

Corresponding author:
Michal Hershfinkel
Department of Physiology and Cell Biology,
Faculty of Health Sciences,
Ben-Gurion University of the Negev,
Beer Sheva, POB 84105, Israel
Tel: +972-8-6477318
hmichal@bgu.ac.il.

**Supplementary figure 1:**

WT colon tissue | ZnR/GPR39 KO colon tissue | WT colon tissue; without 1st Ab

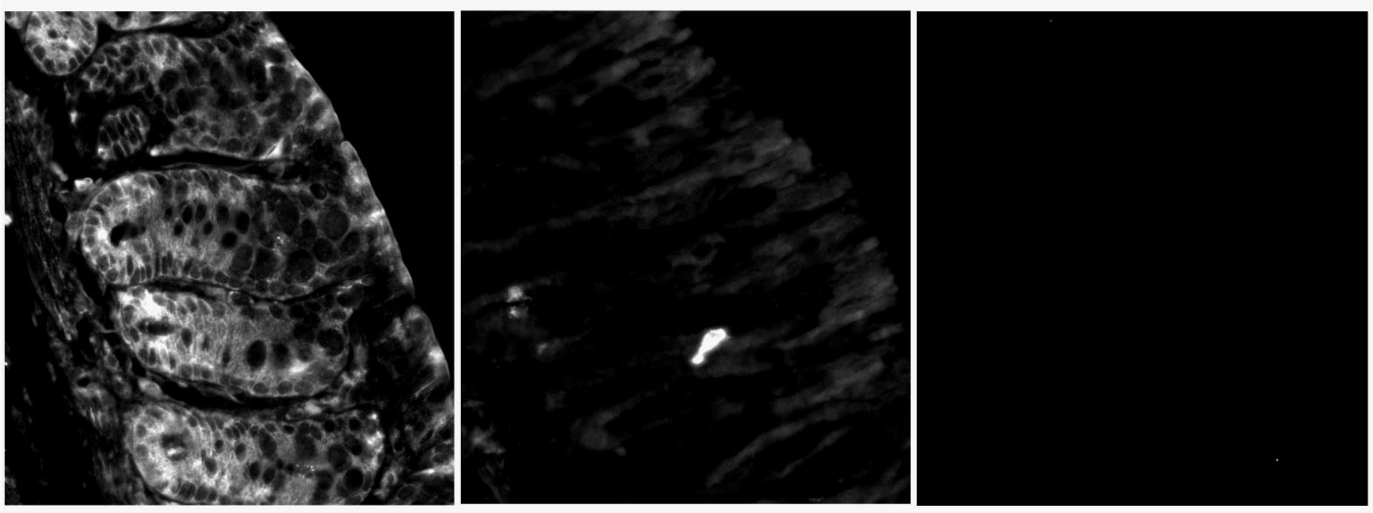

Since the tissue array offered a limited number of tissues we tested the antibody using mouse colon tissue. Colon tissue from WT (right panel) and ZnR/GPR39 KO (center panel) was obtained as described (Sunuwar, Medini, Cohen, Sekler and Hershfinkel in Philos Trans R Soc Lond B Biol Sci. (2016) **371**;1700) ; stained with (right and center panels) or without (right panel) the Abcam GPR39 antibody (Abcam, UK Cat #: AB18859) and imaged using 20X objective, as described in Methods. Clear staining was observed in the WT tissue but only background was seen in the ZnR/GPR39 KO tissue or the control without first antibody.

These complete blots were used in the current study:

**<u>Fig. 5A</u>**

pAKT

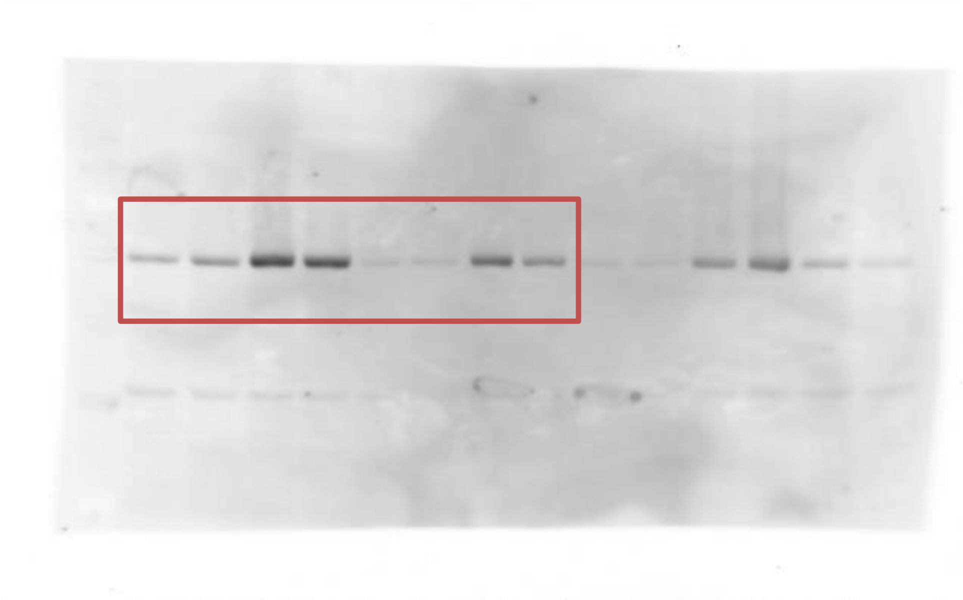

tAKT

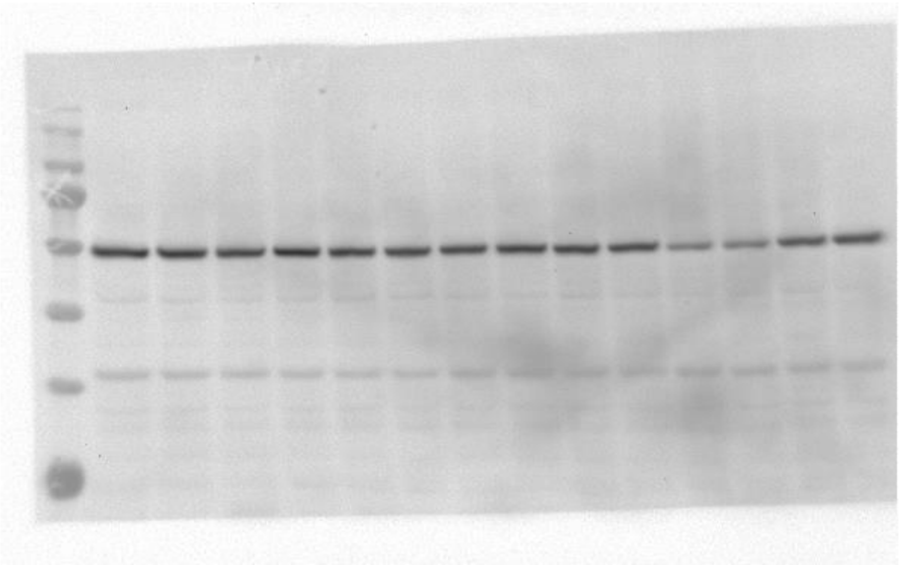

**Fig. 5B**

pAKT

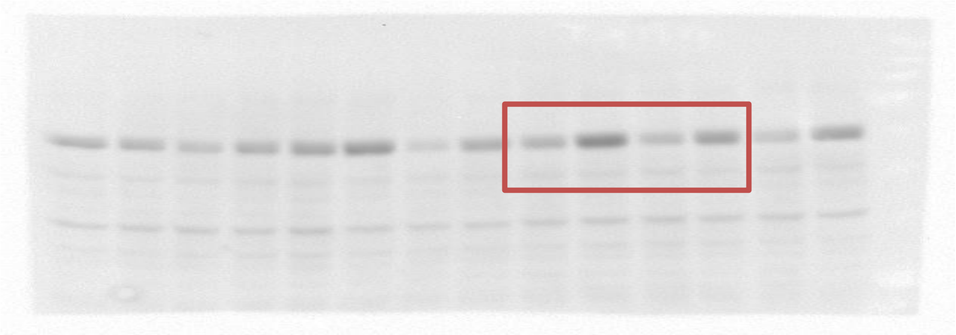

tAKT

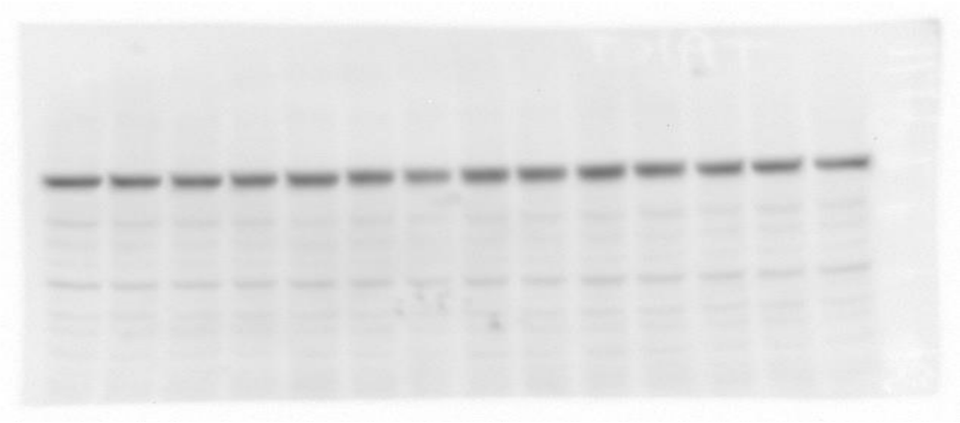

**Fig. 5C**

mTOR

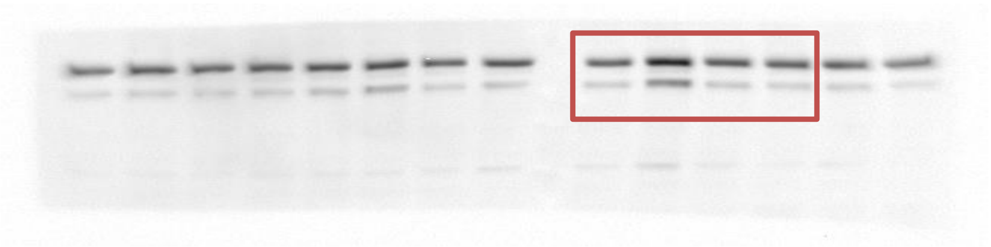

Actin

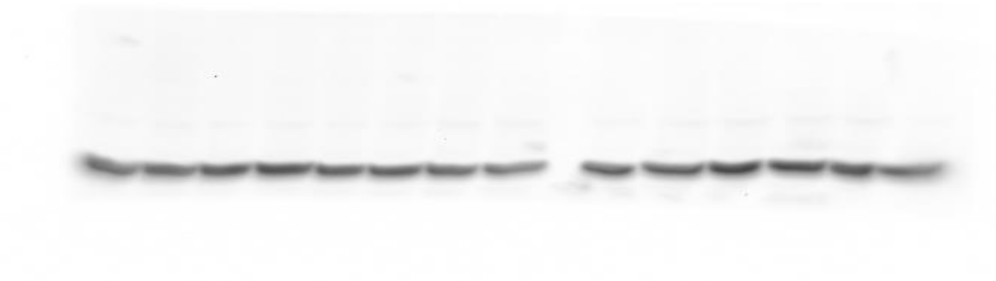

**Fig. 5D**

pERK1/2

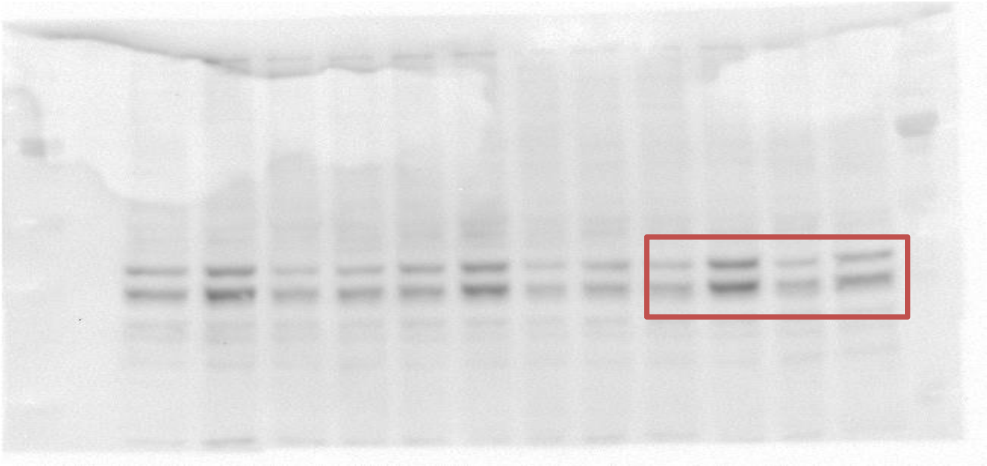

tERK1/2

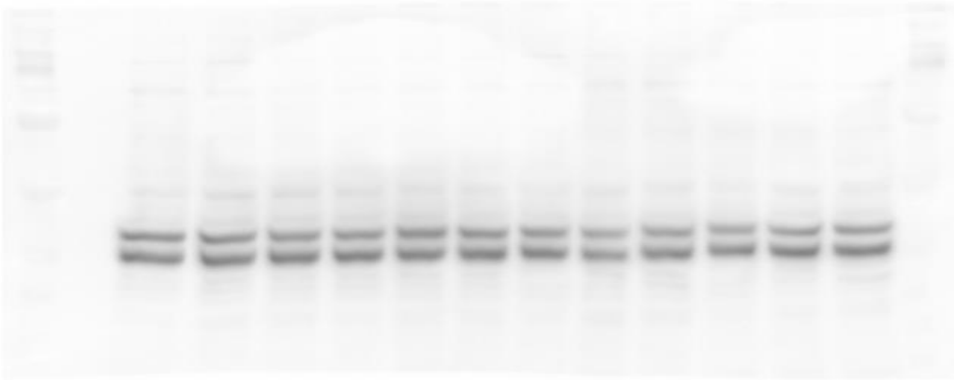

**Fig. 5E**

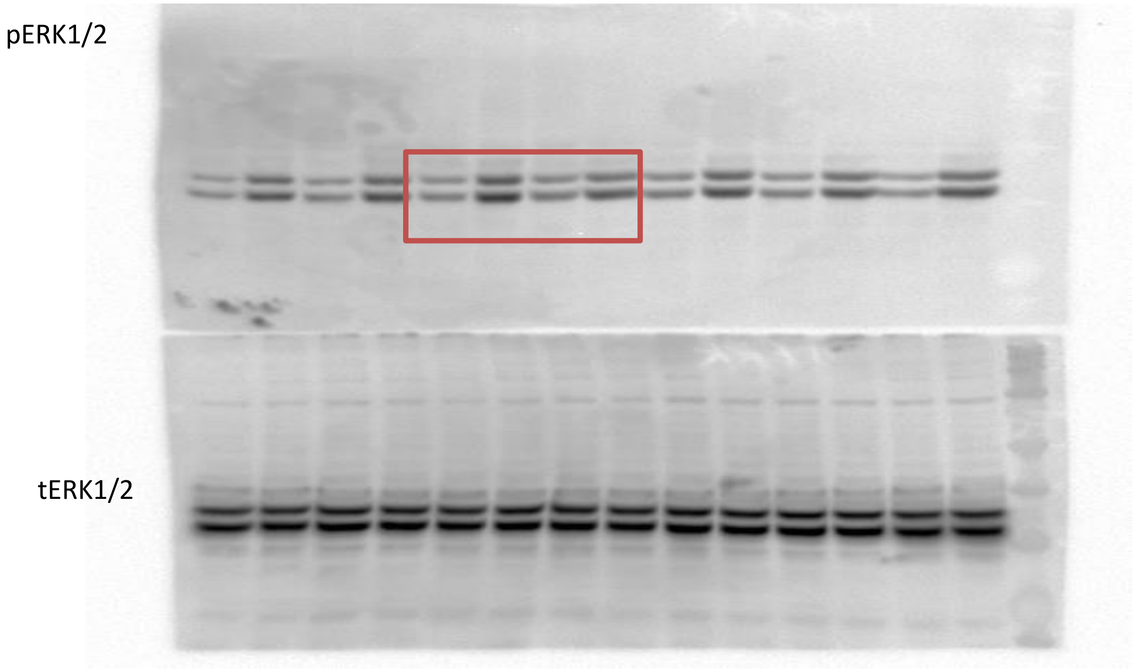

**Fig. 5F**

pERK1/2

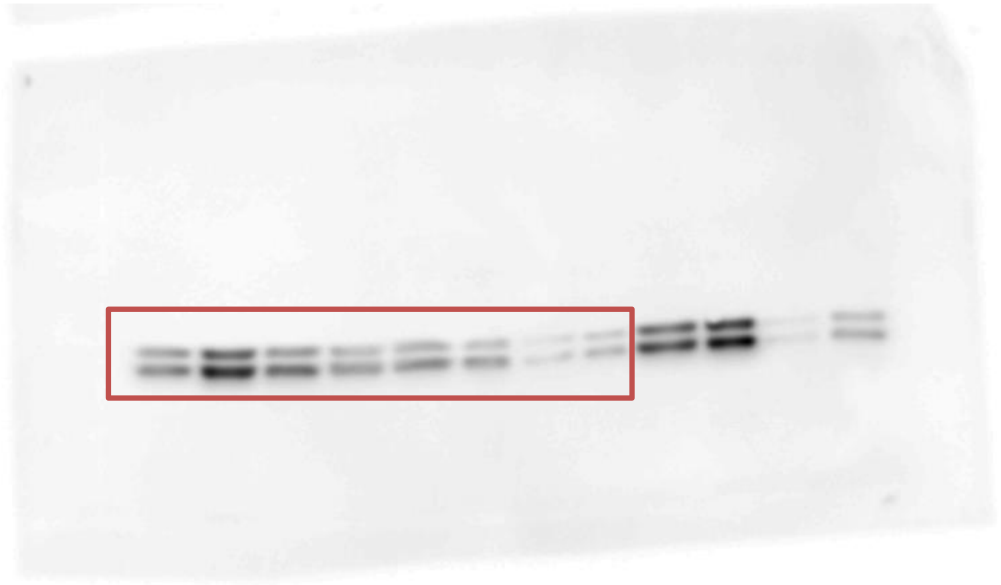

tERK1/2

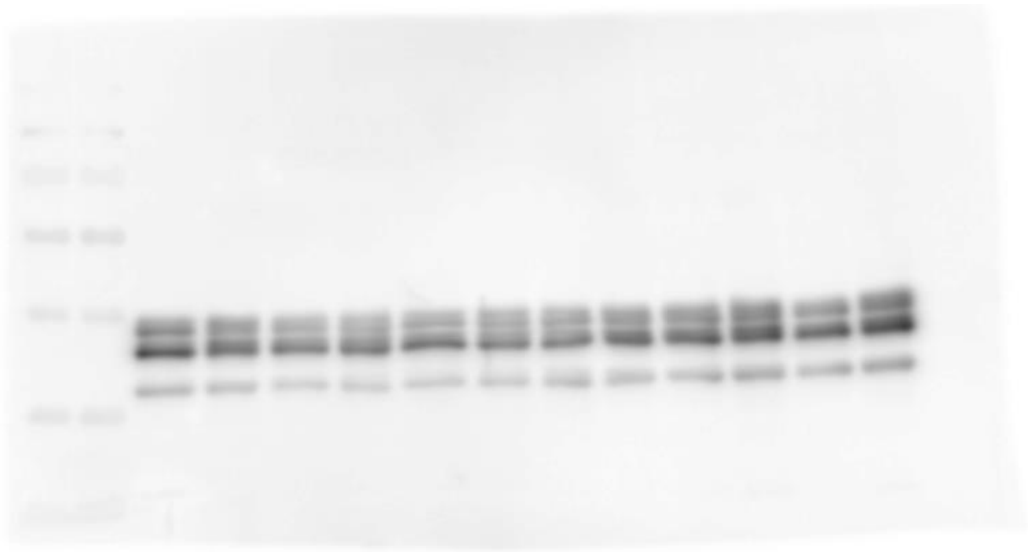

Supplement: Supplementary file 1 — Supplementary Information [file 41598_2018_26459_MOESM1_ESM.pdf]
